# Supplementary material for: Monoclonal Antibody against Porcine LAG3 Inhibits Porcine Reproductive and Respiratory Syndrome Virus Infection
Source: Vet Sci. 2024 Oct 7;11(10):483. doi: 10.3390/vetsci11100483 (PMC11512405; doi:10.3390/vetsci11100483)
Supplement: Supplementary file 1 [file vetsci-11-00483-s001.zip › vetsci-3202864-supplementary.pdf]

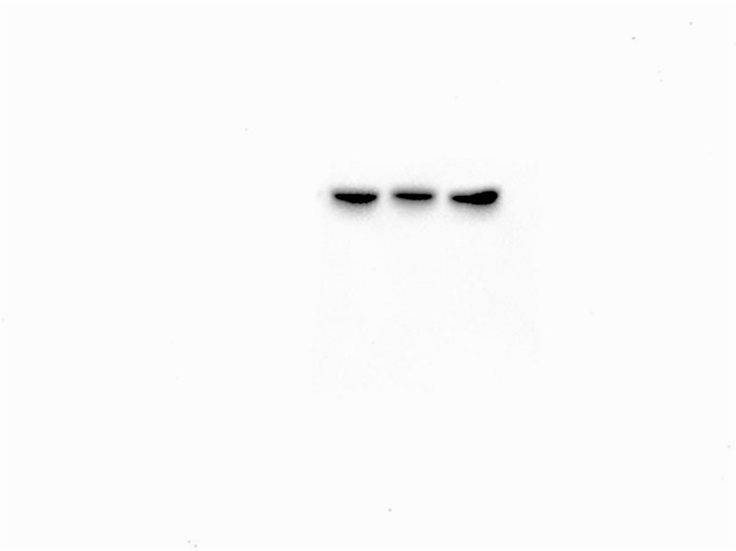

Fig 2B GAPDH left

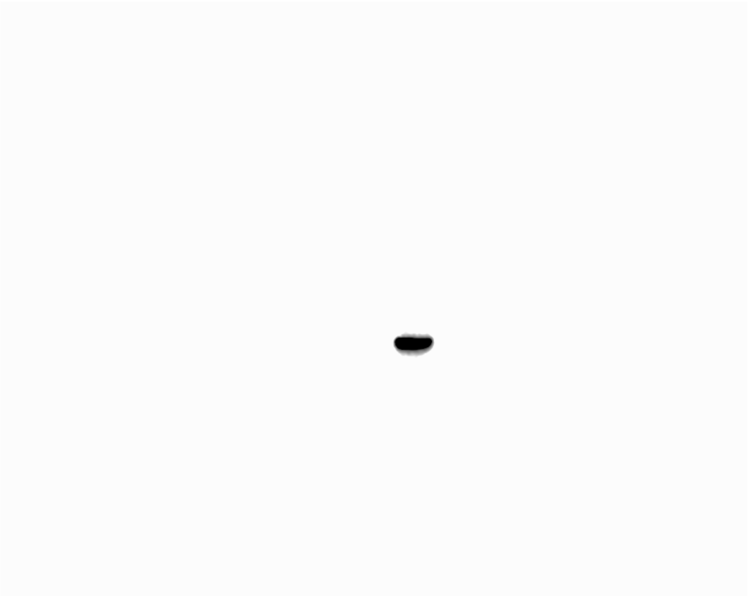

Fig 2B pLAG3 1C2

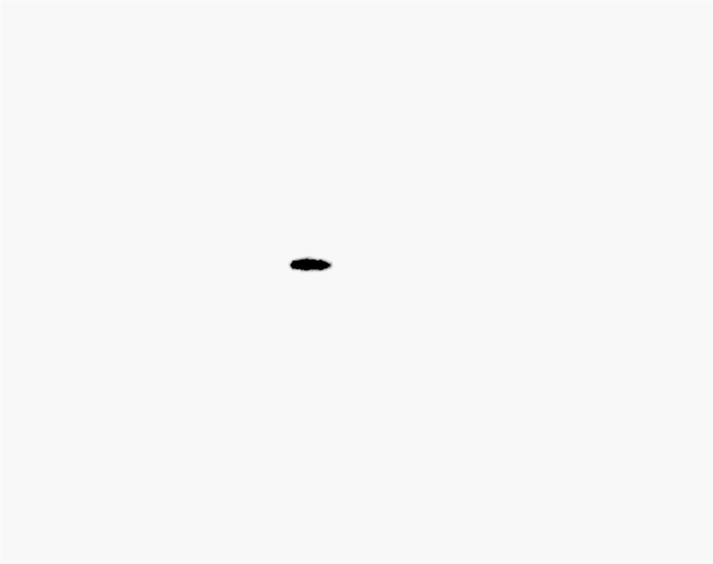

Fig 2B pLAG3 anti-His left

Fig 2B pLAG3 naïve mouse serum left

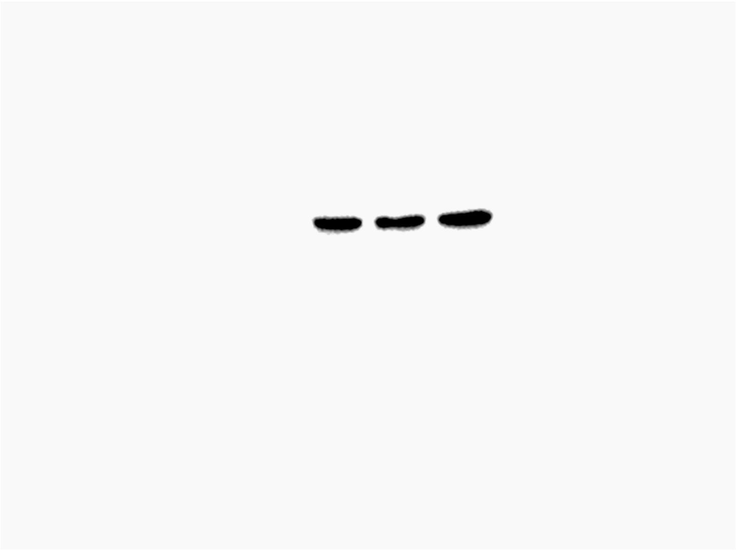

Fig 2B GAPDH right

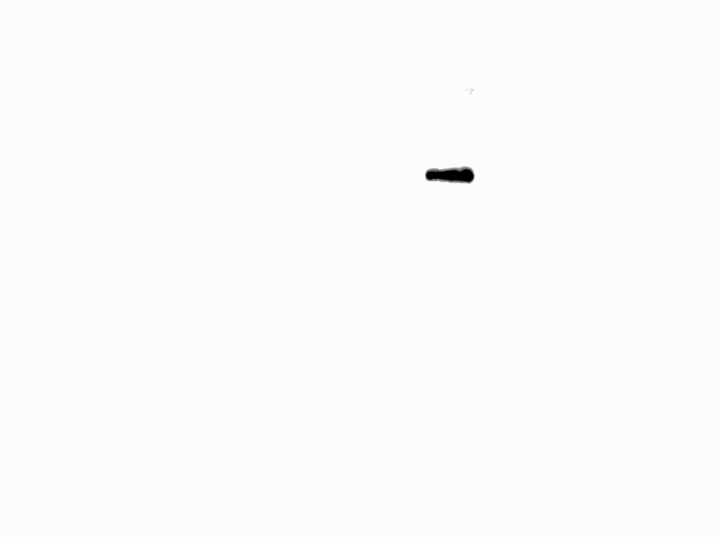

Fig 2B pLAG3 3E11

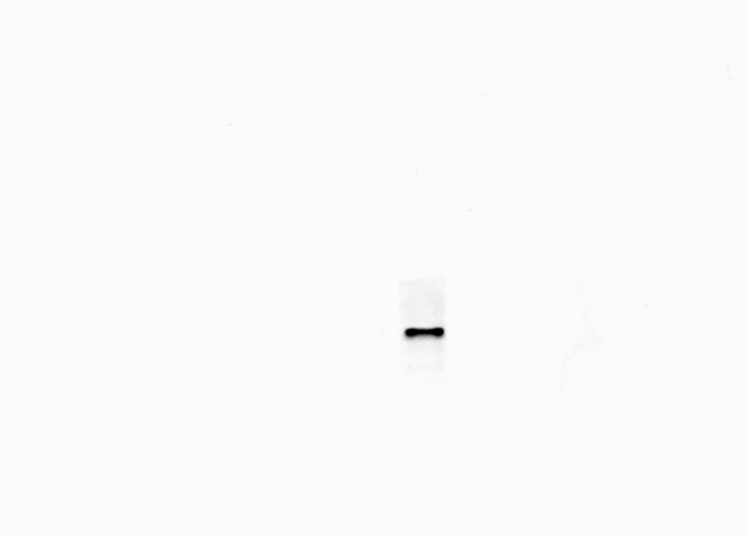

Fig 2B pLAG3 anti-His right

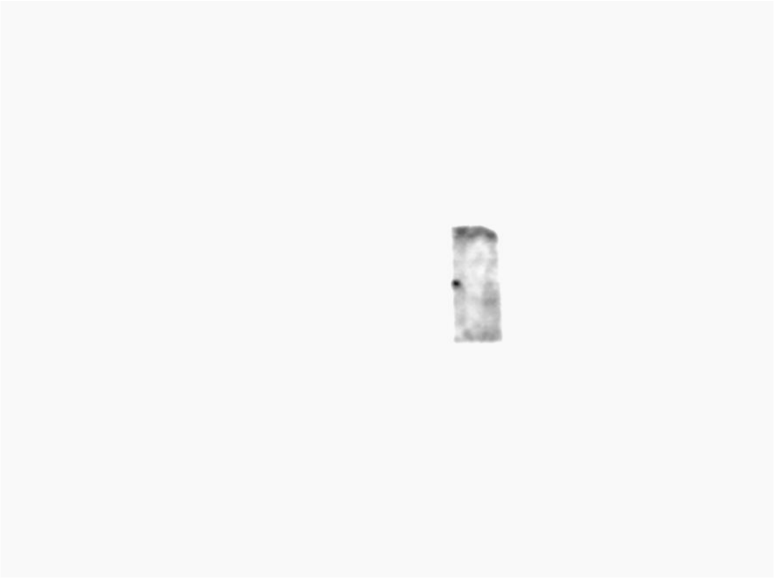

Fig 2B pLAG3 naïve mouse serum right

Figure S1 The original images of Figure 2B

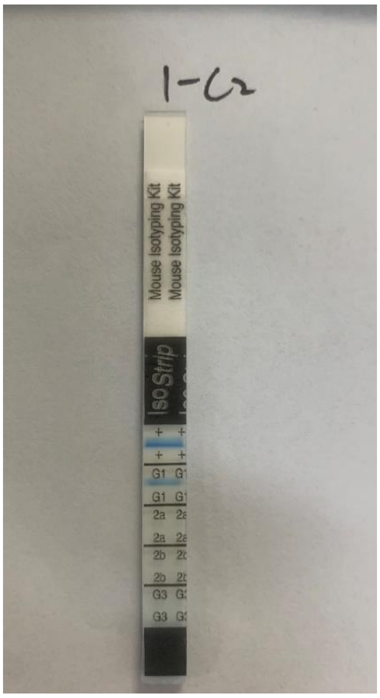

Fig 2E 1C2 heavy chain

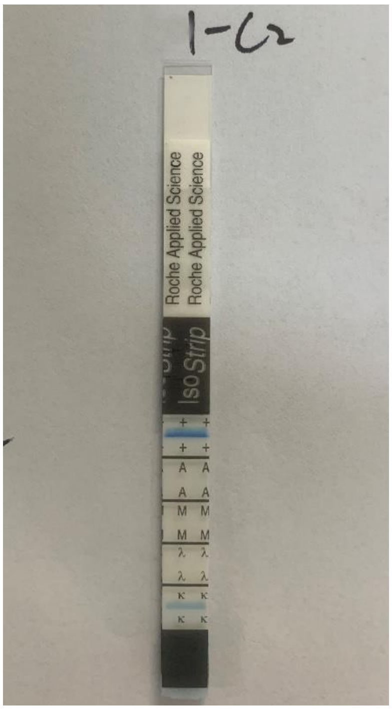

Fig 2E 1C2 light chain

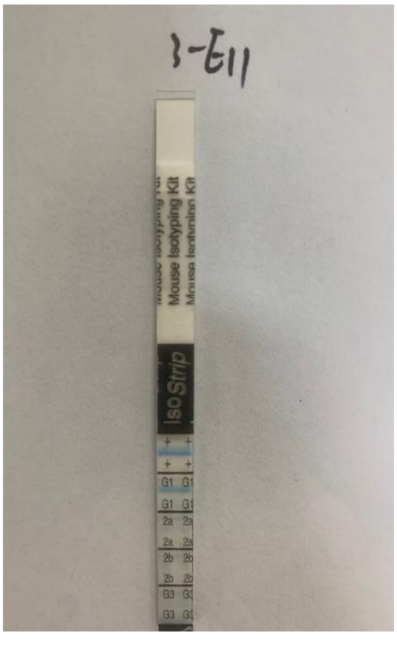

Fig 2E 3E11 heavy chain

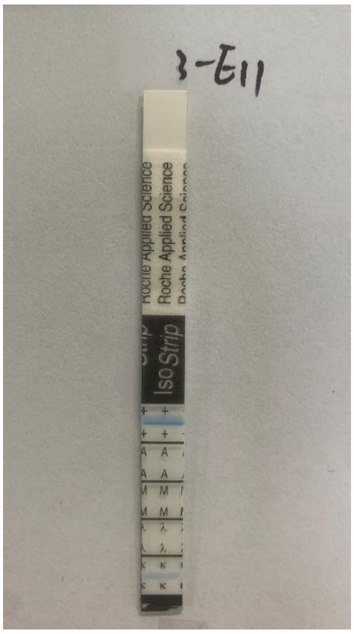

Fig 2E 3E11 light chain

Figure S2 The original images of Figure 2E

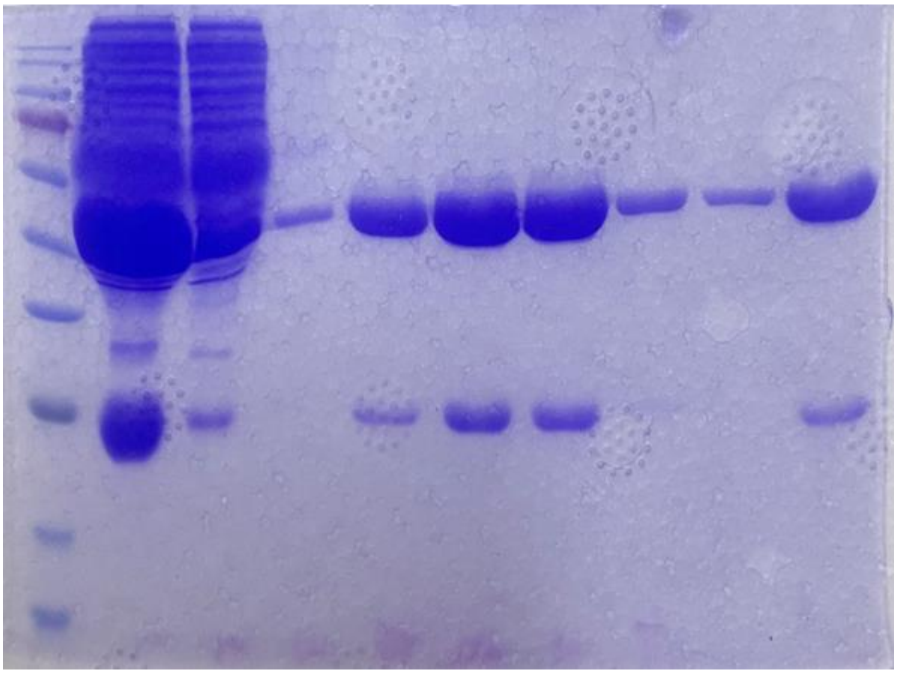

Figure S3 The original images of Figure 2G

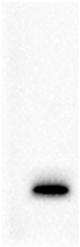

Fig 3B Anti-His HEV-239

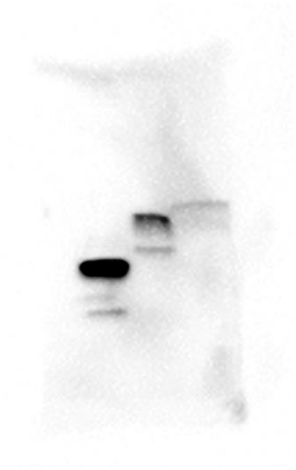

Fig 3B Anti-His pLAG3-2, pLAG3-3

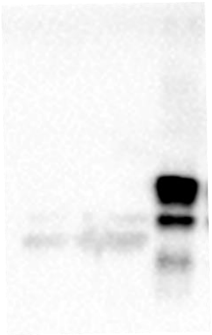

Fig 3B mAb 1C2

Figure S4 The original images of Figure 3B

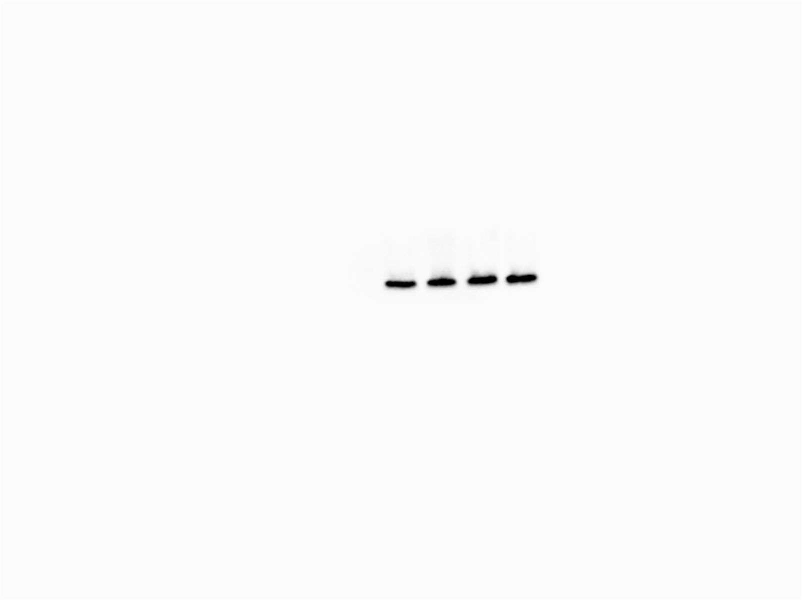

Fig 3C Tubulin

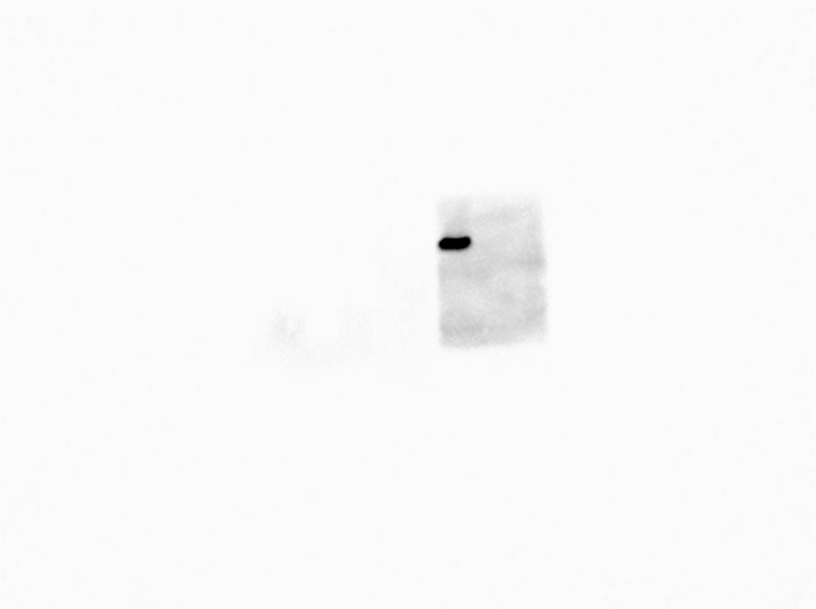

Fig 3C Anti-Flag

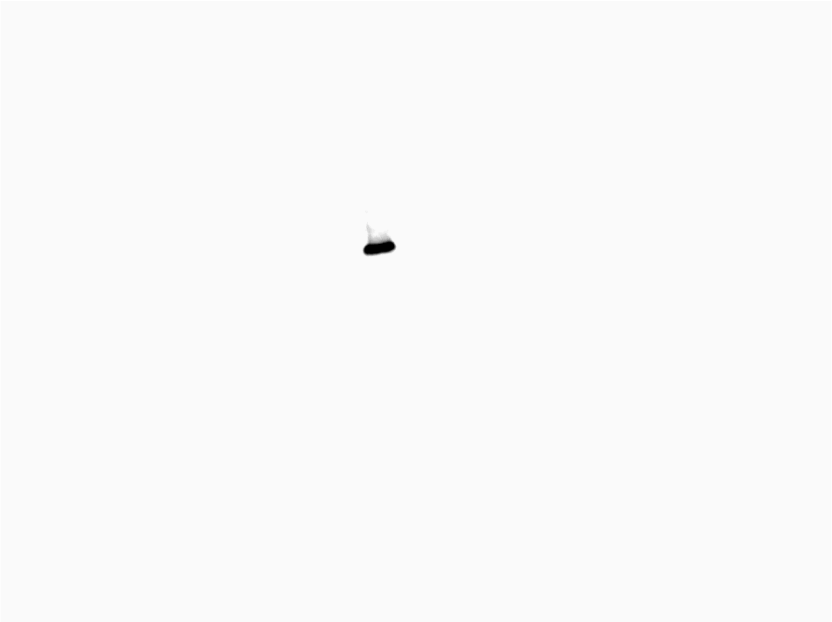

Fig 3C mAb 1C2

Figure S5 The original images of Figure 3C
